# Supplementary material for: Viral priming of cell intrinsic innate antiviral signaling by the unfolded protein response
Source: Nat Commun. 2019 Aug 29;10:3889. doi: 10.1038/s41467-019-11663-2 (PMC6715738; doi:10.1038/s41467-019-11663-2)
Supplement: Supplementary file 1 — Supplementary Information [file 41467_2019_11663_MOESM1_ESM.pdf]

__Supporting Information__

'Viral priming of cell intrinsic innate antiviral signaling by the unfolded protein response' by Tea Carletti, Mohammad Khalid Zakaria et al.

Supplementary Table 1 – Primers' sequences used for real-time qPCR.

Supplementary Figure 1

A, B) Time course of DNAJC3 and DNAJB9 mRNA.

C) Immunofluorescence analysis of ATF6 translocation following TBEV infection.

D) TM and TG treatments inhibit TBEV yields.

E) Effect of TM and TG on TBEV E protein.

F) Induction of Xbp1s following TG treatment.

Supplementary Figure 2

A-F) Tunicamycin treatment of TBEV infected cells led to early ISG induction.

Supplementary Figure 3

A) Efficient depletion of PERK by siRNA.

B) PERK depletion by siRNA did not affect TBEV yields.

C) Blocking the IRE1 RIDD activity affects TBEV-induced Xbp1 splicing.

D) Blocking the IRE1 RIDD activity did not affect TBEV yields.

E – H) Neither ATF6 nor PERK depletion rescued TBEV replication in TM-treated cells.

Supplementary Figure 4

A and B) IRF3 depletion partially rescued WNV replication in TM-treated cells.

C and D) IRF3 depletion partially rescued DENV2 replication in TM-treated cells.

E and F) IRF3 depletion partially rescued ZIKV replication in TM-treated cells. Experiments for ZIKV were conducted as described above for WNV (Supplementary Figure 4 A and B).

G and H) IRE1 depletion could not rescue WNV replication in TM-treated cells.

TBEV 5’ NCR Fw	GCGTTTGCTTCGGA
TBEV 5’NCR Rv	CTCTTTCGACACTCGTCGAGG
β-Actin Fw	CATGTGCAAGGCCGGCTTCG
β-Actin Rv	GAAGGTGTGGTGCCAGATTT
IFNβ Fw	AGGACAGGATGAACTTTGAC
IFNβ Rv	TGATAGACATTAGCCAGGAG
CHOP Fw	TAAAGATGAGCGGGTGGCAG
CHOP Rv	CTGCCATCTCTGCAGTTGGA
XBP1s Fw	CTGAGTCCGCAGCAGGTG
XBP1s Rv	GGCTGGTAAGGAACTGGGTC
XBP1tot Fw	CCGGAGCTGGGTATCTCAAAT
XBP1tot Rv	CCGTATCCACAGTCACTGTAAGCA
DNAJC3 Fw	CGTTTGCGTTCACAAGCACT
DNAJC3 Rv	CCCGAACTTCACTGAGGGAC
DNAJB9 Fw	TGGGGAAGCGTTTCGTGTAG
DNAJB9 Rv	CTAATATCCTGCACCCTCCGAC
BiP Fw	CCCGAGAACACGGTCTTTGA
BiP Rv	TCAACCACCTTGAACGGCAA
IFIT1 Fw	GAAATATGAATGAAGCCCTGGA
IFIT1 Rv	GACCTTGTCTCACAGAGTTCTCAA
Viperin Fw	ATGGAGTTGACATGGAGGCA
Viperin Rv	GCTCACATTCTTCCTTGCCC
IFNβ mouse Fw	GCACTGGGTGGAATGAGACT
IFNβ mouse Rv	AGTGGAGAGCAGTTGAGGACA
β-Actin mouse Fw	GGCACCACACCTTCTACAATG
β-Actin mouse Rv	GTGGTGGTGAAGCTGTAGCC
IFI44L Fw	TCAAAGCCGGGTCATGAATG
IFI44L Rv	CCTTCATGGGGTCCAGTTCC
IFI44 Fw	AGACGAATGCTATGGGCTGC
IFI44 Rv	CCTCCCTTAGATTCCCTATTTGCTC
Viperin (second set) Fw	CCCCAACCAGCGTCAACTAT
Viperin (second set) Rv	TTGATCTTCTCCATACCAGCTTCC
IFIH1 Fw	GATTCAGGCACCATGGGAAGT
IFIH1 Rv	AGGCCTGAGCTGGAGTTCTG
OASL Fw	TACCAGTATGTGAAAGCCA
OASL Rv	GGTGAAGCCTTCGTCCAACA

Supplementary Table 1 – Primers’ sequences used for real-time qPCR.

The Table shows the sequence of the primers (forward and reverse) used for real-time qPCR of the indicated genes and viral sequences.

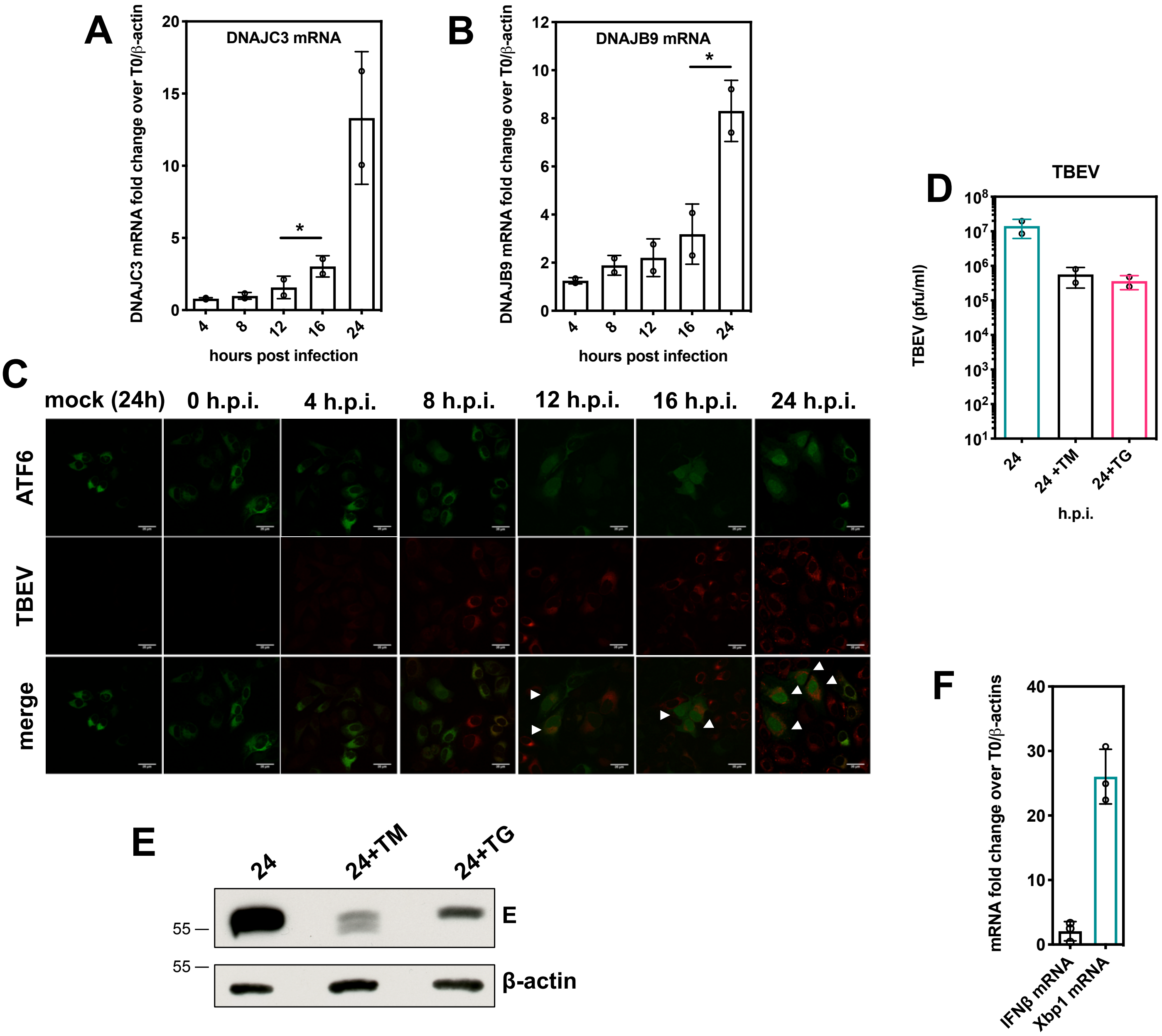

Supplementary Figure 1.

A, B) Time course of DNAJC3 and DNAJB9 mRNA. U2OS cells were infected with TBEV as in Figure 1. Total RNA was extracted at the indicated time points and used as template for qPCR using specific primers. TBEV RNA amplification products were normalized to β-actin RNA and plotted as fold-change from time 0. Average values of triplicate independent experiments are shown, with standard deviations and *P* values, as described in the Methods section.

C) Immunofluorescence analysis of ATF6 translocation following TBEV infection. U2OS cells were transfected with EGFP-ATF6 and the following day infected with TBEV at moi = 1. At the indicated time point cells were fixed and stained for the TBEV E protein AlexaFluor 594, red). White arrowheads indicate translocated ATF6. Scale bar 20 µm.

D) TM and TG treatments inhibit TBEV yields. U2OS cells were either infected with TBEV (blue bar) or treated with TM (black bar) or TG (magenta bar) immediately after infection. Supernatants from infected cells were used to infect Vero cells to measure virus yields (PFU/ml). Average values of duplicate independent experiments are shown.

E) Effect of TM and TG on TBEV E protein. U2OS infected as in Supplementary Figure 1D above were lysed 24 hpi and blotted for TBEV E protein and for β-actin as loading control.

F) Induction of Xbp1s following TG treatment. U2OS cells were treated for 8 hours with TG 0.5 µM and Xbp1s/ IFNβ mRNA measured by qPCR as above (A).

Source data are provided as a Source Data file.

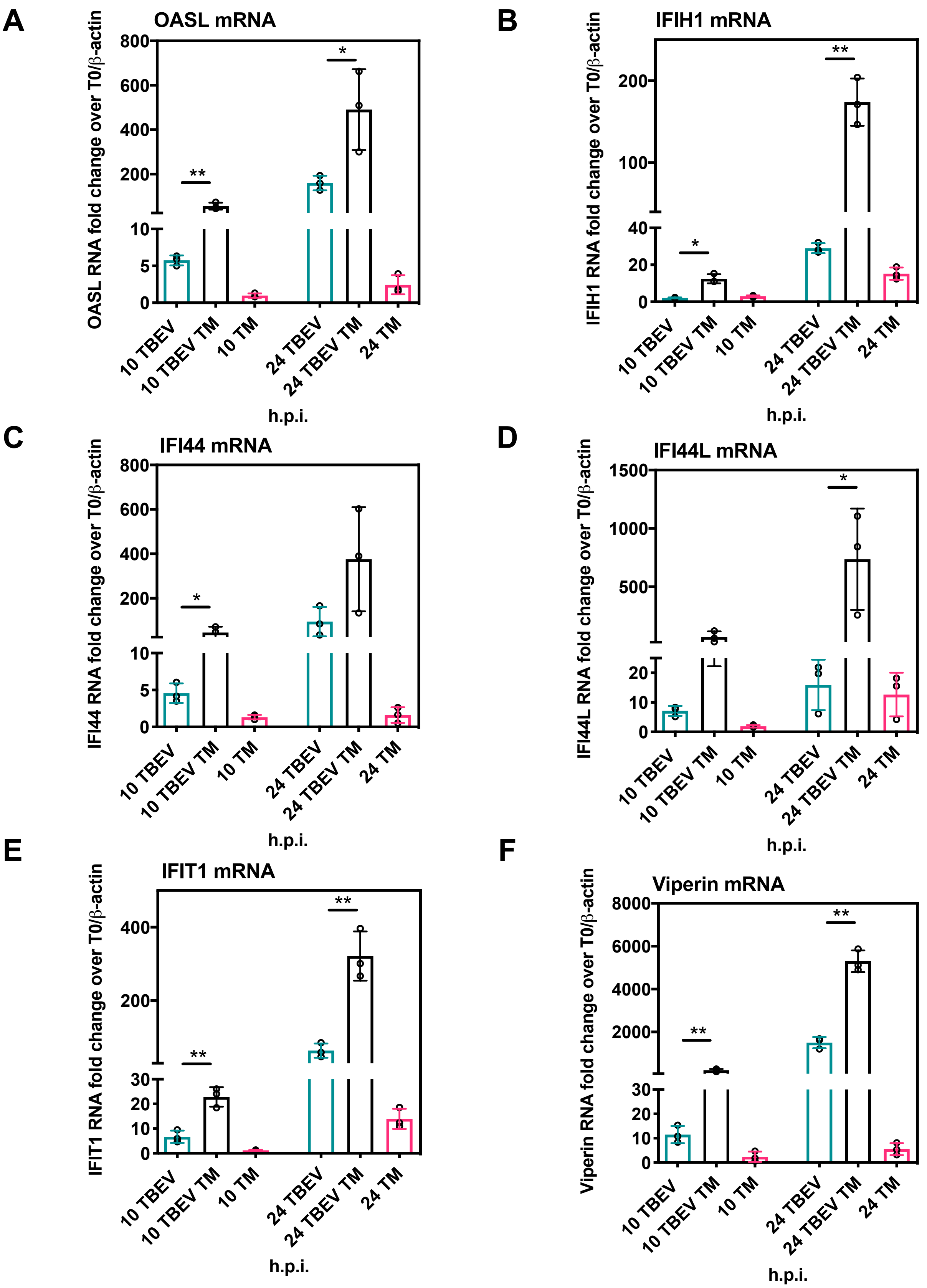

Supplementary Figure 2

A-F) <u>Tunicamycin treatment of TBEV infected cells led to early ISG induction</u>.

Total RNA from U2OS cells infected with TBEV moi = 1 was extracted at the indicated time points and used as template for real-time qPCR using primers specific for OASL, IFIH1, IFI44, IFI44L, IFIT1 or Viperin (second set). Values of ISG mRNA from mock-infected cells treated with Tunicamycin were also indicated (magenta bars). Average values of triplicate independent experiments are shown as described in the Methods section.

Source data are provided as a Source Data file.

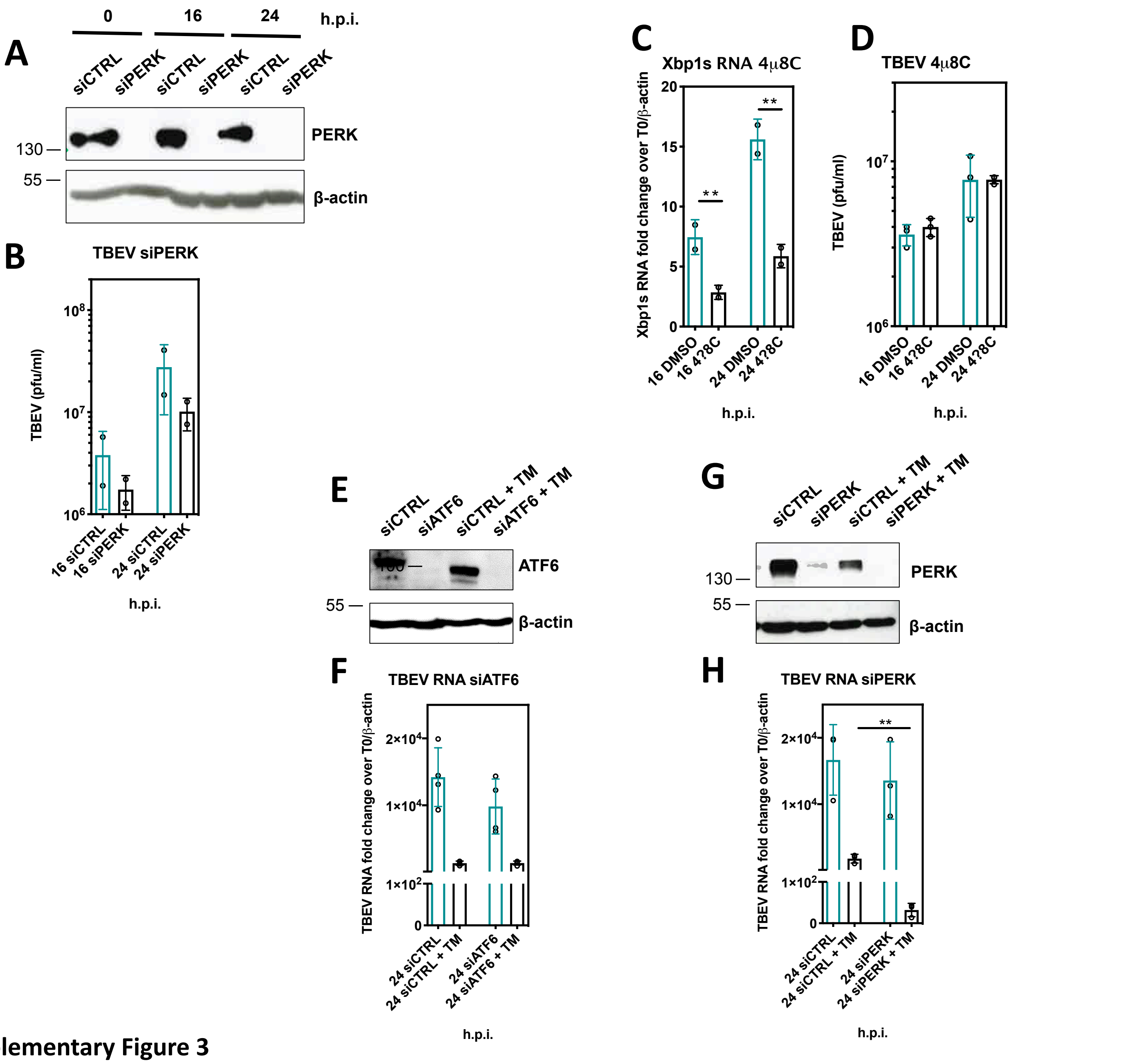

Supplementary Figure 3

A) Efficient depletion of PERK by siRNA. U2OS cells were transfected with siPERK/siCTRL and after 48 hours infected with TBEV. Cells were harvested for immunoblotting at 16 and 24 h.p.i. Actin is the protein loading control.

B) PERK depletion by siRNA did not affect TBEV yields. U2OS cells depleted for PERK as above were infected with TBEV at moi = 1. At the indicated time points, supernatants from infected cells were used to infect Vero cells to measure virus yields (pfu/ml).

C) Blocking the IRE1 RIDD activity affects TBEV-induced Xbp1 splicing. U2OS cells were infected with TBEV and treated with 30 µM 4µ8C. Total RNA extracted at the indicated time points was used as template for real-time qPCR with primers specific for Xbp1s mRNA. TBEV RNA amplification products were normalized to β-actin RNA and plotted as fold-change from time 0.

D) Blocking the IRE1 RIDD activity did not affect TBEV yields. U2OS cells were infected with TBEV and treated with 30 µM 4µ8C. Supernatants from infected cells at the indicated time points were used to infect Vero cells to measure virus yields (pfu/ml). Average values of triplicate independent experiments are shown with standard deviations and *P* values as described in the Methods section.

E – H) Neither ATF6 nor PERK depletion rescued TBEV replication in TM-treated cells. U2OS cells were either infected with TBEV (blue bars) or treated with TM immediately after infection (black bars) in conditions of ATF6 or PERK depletion. Immunoblot for ATF6/PERK and TBEV RNA quantification was performed 24 h.p.i. Average values of triplicate independent experiments are shown as described in the Methods section.

Source data are provided as a Source Data file.

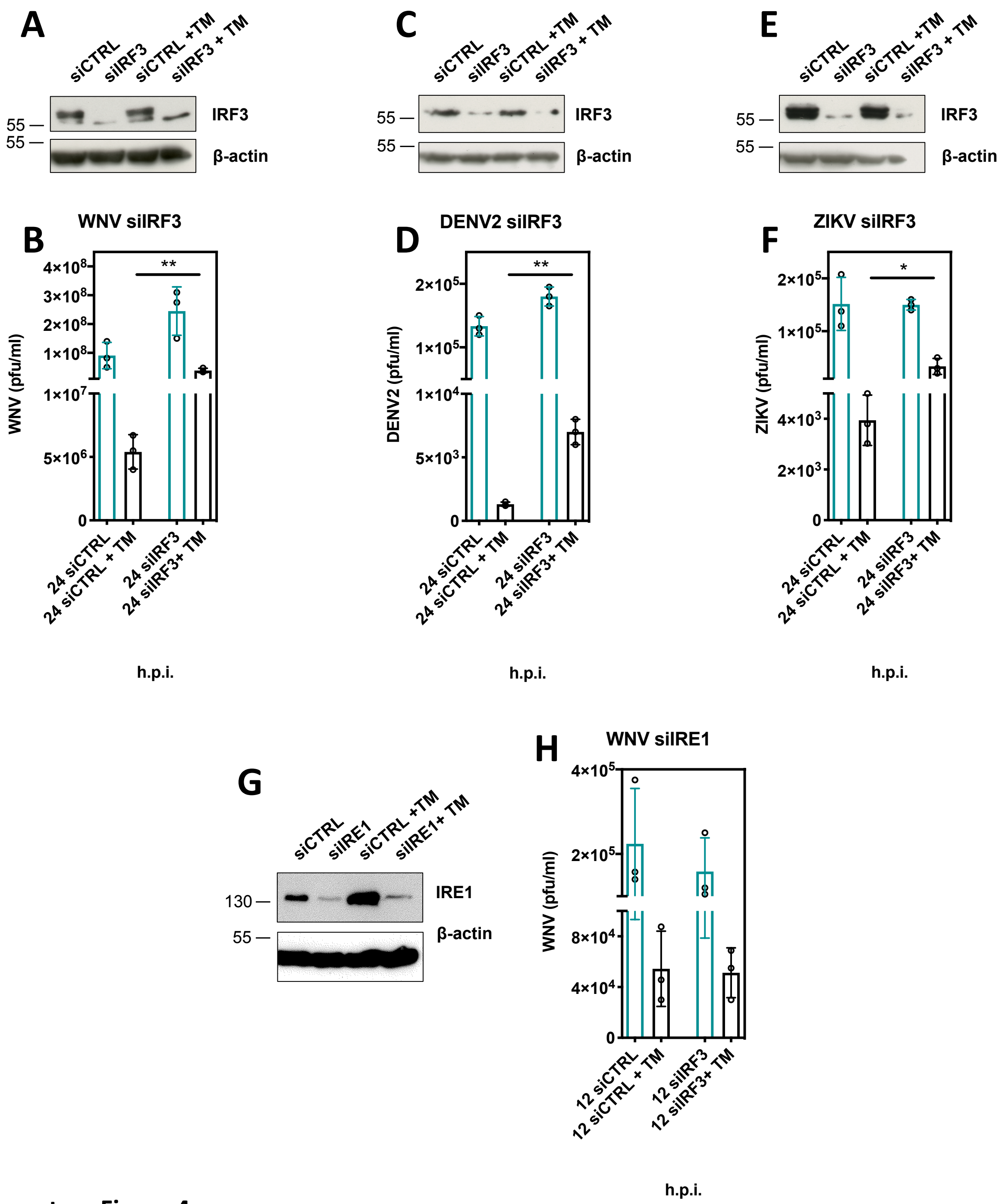

Supplementary Figure 4

A and B) IRF3 depletion partially rescued WNV replication in TM-treated cells. U2OS cells were either infected with WNV (blue bars) or treated with TM immediately after infection (black bars) in conditions of IRF3 depletion. Immunoblot for IRF3 and WNV RNA quantification was performed 24 h.p.i. Average values of triplicate independent experiments are shown as described in the Methods section.

C and D) IRF3 depletion partially rescued DENV2 replication in TM-treated cells. Experiments for DENV2 were conducted as described above for WNV (Supplementary Figure 4 A and B).

E and F) IRF3 depletion partially rescued ZIKV replication in TM-treated cells. Experiments for ZIKV were conducted as described above for WNV (Supplementary Figure 4 A and B).

G and H) IRE1 depletion could not rescue WNV replication in TM-treated cells. U2OS cells were either infected with WNV (blue bars) or treated with TM immediately after infection (black bars) in conditions of IRE1 depletion. Immunoblot for IRE1 and WNV RNA quantification was performed 12 h.p.i. Average values of triplicate independent experiments are shown as described in the Methods section.

Source data are provided as a Source Data file.
