# Supplementary material for: Viral priming of cell intrinsic innate antiviral signaling by the unfolded protein response
Source: Nat Commun. 2019 Aug 29;10:3889. doi: 10.1038/s41467-019-11663-2 (PMC6715738; doi:10.1038/s41467-019-11663-2)
Supplement: Supplementary file 3 — Reporting Summary [file 41467_2019_11663_MOESM3_ESM.pdf]

nature research

Corresponding author(s): Alessandro Marcello

Last updated by author(s): Jul 10, 2019

Reporting Summary

Nature Research wishes to improve the reproducibility of the work that we publish. This form provides structure for consistency and transparency in reporting. For further information on Nature Research policies, see Authors & Referees and the Editorial Policy Checklist.

Statistics

For all statistical analyses, confirm that the following items are present in the figure legend, table legend, main text, or Methods section.

n/a	Confirmed	
☐	☒	The exact sample size (*n*) for each experimental group/condition, given as a discrete number and unit of measurement
☐	☒	A statement on whether measurements were taken from distinct samples or whether the same sample was measured repeatedly
☐	☒	The statistical test(s) used AND whether they are one- or two-sided<br>*Only common tests should be described solely by name; describe more complex techniques in the Methods section.*
☒	☐	A description of all covariates tested
☒	☐	A description of any assumptions or corrections, such as tests of normality and adjustment for multiple comparisons
☐	☒	A full description of the statistical parameters including central tendency (e.g. means) or other basic estimates (e.g. regression coefficient) AND variation (e.g. standard deviation) or associated estimates of uncertainty (e.g. confidence intervals)
☐	☒	For null hypothesis testing, the test statistic (e.g. *F*, *t*, *r*) with confidence intervals, effect sizes, degrees of freedom and *P* value noted<br>*Give P values as exact values whenever suitable.*
☒	☐	For Bayesian analysis, information on the choice of priors and Markov chain Monte Carlo settings
☒	☐	For hierarchical and complex designs, identification of the appropriate level for tests and full reporting of outcomes
☒	☐	Estimates of effect sizes (e.g. Cohen's *d*, Pearson's *r*), indicating how they were calculated

Our web collection on statistics for biologists contains articles on many of the points above.

Software and code

Policy information about availability of computer code

Data collection: *Provide a description of all commercial, open source and custom code used to collect the data in this study, specifying the version used OR state that no software was used.*

Data analysis: *Provide a description of all commercial, open source and custom code used to analyse the data in this study, specifying the version used OR state that no software was used.*

For manuscripts utilizing custom algorithms or software that are central to the research but not yet described in published literature, software must be made available to editors/reviewers. We strongly encourage code deposition in a community repository (e.g. GitHub). See the Nature Research guidelines for submitting code & software for further information.

Data

Policy information about availability of data

All manuscripts must include a data availability statement. This statement should provide the following information, where applicable:

- Accession codes, unique identifiers, or web links for publicly available datasets
- A list of figures that have associated raw data
- A description of any restrictions on data availability

Data underlying Figure 1 B to J, Figure 2 A to L, Figure 3 A to C and E to H, Figure 4 A to N, Supplementary Figure 1 A, B and D to F, Sup. Figure 2 A to F, Sup. Figure 3 A to H; Sup. Figure 4 A to H are provided as Source Data files. All other data are available from the corresponding author upon reasonable requests. RNAseq data have been deposited with links to BioProject accession number PRJNA474353 in the NCBI BioProject database [https://www.ncbi.nlm.nih.gov/bioproject/]. Sequence Read Archive (SRA) submission: SUB4111543 - 1st of June 2018, SRA accession: SRP149625.

Field-specific reporting

Please select the one below that is the best fit for your research. If you are not sure, read the appropriate sections before making your selection.

☒ Life sciences ☐ Behavioural & social sciences ☐ Ecological, evolutionary & environmental sciences

For a reference copy of the document with all sections, see nature.com/documents/nr-reporting-summary-flat.pdf

Life sciences study design

All studies must disclose on these points even when the disclosure is negative.

Sample size	Typically, three biologically independent experiments (n=3) in triplicate repeats were conducted for each condition examined, unless otherwise specified. This is a standard sample for the kind of experiments described in the paper used in life sciences.
Data exclusions	No data were excluded from the analysis
Replication	All experiments were reproducible at least in three biologically independent repeats
Randomization	Allocation of samples was random
Blinding	Investigators were blinded to groups' allocation when performing manual counting from micrographs (Figure 3E)

Reporting for specific materials, systems and methods

We require information from authors about some types of materials, experimental systems and methods used in many studies. Here, indicate whether each material, system or method listed is relevant to your study. If you are not sure if a list item applies to your research, read the appropriate section before selecting a response.

Materials & experimental systems

n/a	Involved in the study	
☐	☒	Antibodies
☐	☒	Eukaryotic cell lines
☒	☐	Palaeontology
☒	☐	Animals and other organisms
☒	☐	Human research participants
☒	☐	Clinical data

Methods

n/a	Involved in the study	
☒	☐	ChIP-seq
☒	☐	Flow cytometry
☒	☐	MRI-based neuroimaging

Antibodies

Antibodies used	The following antibodies were used in this study: a rabbit polyclonal against the TBEV E protein produced in our laboratory following immunization with the inactivated virions (1:100 IF, 1:1000 WB); a rabbit polyclonal against TBEV prM kindly provided by Franz Heinz, Vienna (1:100 WB); a rabbit polyclonal against human eIF2α from SCBT (1:100 WB, cat.no. sc-11386); a rabbit polyclonal against phosphorylated (Ser51) human eIF2α from Cell Signaling (1:500 WB, cat.no. 9721); a rabbit polyclonal against human PERK from SCBT (1:500 WB, cat.no. sc-13073); a rabbit monoclonal against human PERK from Cell Signaling (1:1000 WB, cat.no. 3192); a rabbit polyclonal against phosphorylated (T981) human PERK from SCBT (1:200 WB, cat.no. sc-32577); a mouse monoclonal against human PKR from SCBT (1:200 WB, cat.no. sc-6282); a mouse monoclonal against human β-catenin from BD Transduction Lab (1:2000 WB, cat.no. 610153); a mouse monoclonal against human β-actin conjugated with peroxidase from Sigma-Aldrich (1:50000 WB, A3854); a rabbit monoclonal against IRF3 from Cell Signaling (1:1000 WB, cat.no. 4302); a mouse monoclonal against dsRNA from English & Scientific Consulting (1:100 IF, J2-1101) a rabbit monoclonal against phospho-IRF3 from Cell Signaling (1:500 WB, cat.no. 4947); a mouse monoclonal against ATF6 from Abcam (1:500 WB, cat.no. ab122897); a rabbit monoclonal against IRE1 from Cell Signaling (1:1000 WB, cat.no. 3294); a mouse monoclonal against RIG-I from Adipogen (WB 1:500, AG-20B-0009). Secondary antibodies conjugated with AlexaFluor 488/594 were from Life Technologies (1:500 IF, cat.no. anti-mouse 594 A21207 and anti-rabbit 488 A-21206) and peroxidase conjugates from Dako (1:5000 WB, cat.no. anti-rabbit P0448 and anti-mouse P0447).
Validation	Rabbit polyclonal against the TBEV E protein produced in our laboratory following immunization with the inactivated virions was validated in house for Western blot and IF using reference material; Rabbit polyclonal against TBEV prM kindly provided by Franz Heinz, Vienna and validated by them and in house for Western blot and IF using reference material; Commercial antibodies were validated by the manufacturers and were used previously by the group or by the scientific community for similar studies.

Eukaryotic cell lines

Policy information about cell lines

Cell line source(s)	Vero clone E6 (ATCC C1008) and human osteosarcoma U2OS cells (ATCC HTB-96)
Authentication	Cell lines used in this work are standard cells obtained by referenced sources maintained in the laboratory under controlloed passaging conditions and were not further authenticated.
Mycoplasma contamination	All cell lines were routinely tested for Mycoplasma contamination. Cells use for the reported experiments were free of contamination and passaged without antibiotics.
Commonly misidentified lines (See ICLAC register)	none
